# Institutional Notes and News

**Published:** 1910-12-24

**Authors:** 


### Institutional Notes and News.

**The London Homœopathic Hospital.**

The London Homœopathic Hospital is now engaged in a race against time. Before December 31 £2,000 have to be secured if the Hospital is not to lose a promise of £5,000. Our readers will remember that we called attention to this promise last March, when, on page 733 of our issue of the 19th of that month, a conditional gift of £5,000 from Lord Dysart was mentioned. If this is gained, as by the public's intelligence and generosity it can be, the new Sir Henry Tyler Wing and the Nurses' Home will have been paid for without any encroachment on the reserve fund of the Hospital. What is more, public subscription to-day will prevent any modification of the original plan for the new wing, and that means that the necessary alterations will be completed, so that the London Homœopathic Hospital may start the New Year with its house in order. Modification generally means postponement, and money is saved by carrying out at one time any structural alterations and extensions that are necessary. Generosity and economy in this case are interdependent. We wish the appeal all success.

**St. John's Hospital, Lewisham.**

Blackheath, Deptford, Greenwich, Catford, and Lee, consciously or unconsciously, are awaiting the result of the Christmas Appeal which St. John's Hospital, Lewisham, has just issued. All these great suburbs send relays of out-patients to the hospital, and that being so it is not surprising to hear that the out-patient department needs extension. At present efforts are being made to see what rearrangement can do at this busy time of year, but a new building is needed of up-to-date design, where the out-patients not only can be received but in some degree sorted, for the great principle of classification is being recognised in every social department of modern life, and the principle which is being applied to school children is also in process of being perfected in the hospitals. These grounds, social and intelligent, rather than sentimental, are quite sufficient to attract the notice of all public-spirited givers, and we prefer to emphasise the hospital's claims in this respect than even to remind our readers that for two months this year the hospital had to be closed.

**North Lonsdale Hospital.**

Mr. F. S. Ramsden, vice-president, took the chair at the recent annual meeting of the North Lonsdale Hospital, Barrow-in-Furness. The hospital begins its New Year with a balance of £114, which is being carried over. It was, however, not merely the annual report which the meeting was asked to adopt, but a special report also, which the hospital's committee has issued to subscribers, and which deals with certain allegations that have been appearing in the local papers. This report should inspire the subscribers with confidence, for it states that the complaints have been investigated, and the alleged breach of duties disproved, while it adds, very forcibly, that the committee is prepared at all times to consider complaints, criticisms or grievances, but those who make them should make them, in everyone's interest, at the time, and not six months after the matter is made semi-incapable of investigation. Mr. E. S. Major remarked that the balance being carried over ought to have been a great deal larger; at present they did not have enough to create a sinking fund.

**Cardiff and the Welsh Memorial.**

Mr. David Davies, M.P., who has subscribed £25,000 to the Welsh King Edward Memorial Fund, Lord Kenyon, and Mr. Thomas Jones (secretary) conferred with the Executive of the West Wales Sanatorium on Monday at Carmarthen to find out how the founders of the Alltymynydd Sanatorium, of which Princess Christian is patron, propose to swell the National Memorial subscriptions. These, we learn, are coming in slowly. Three hundred thousand pounds are required to endow and to establish dispensaries throughout the kingdom, and to give the education which ultimately will eradicate consumption. Readers of The Hospital will remember that an important movement is on foot to associate the financial resuscitation of Cardiff Infirmary with the Welsh National Memorial. The arguments, of which Colonel Bruce Vaughan has been the chief spokesman, in favour of this policy, are so convincing that they have gained considerable support in Cardiff, and all public-spirited citizens will see that the Christmas holidays do not interfere with their advocacy. Want of space prevents us from repeating them here, but they will be found by turning to the files of The Hospital for October 22, November 26, on pages 119 and 269 respectively.

**Derbyshire Children's Hospital.**

Mr. F. W. Graves, J.P., in the absence of the President, the Hon. Blanche Curzon, took the chair at the annual meeting of the Derbyshire Hospital for Sick Children. He said the additions, including the nursing home, were justified fully; indeed, during the past four months thirty-five children had been waiting admission. The deficiency on the year's work is one of £976, which would have been greater had not the lady collectors, and the ex-President, Miss Curzon, worked so vigorously. The Children's Hospital at Derby, as the fact that its past three presidents have been ladies shows, relies largely on the energy and enthusiasm of women workers, not only on its institutional but also on its medical side. The house surgeon for the year is Miss Annie E. Greenep, M.B., C.M., who succeeded Miss M. H. Archibald last July. The number of in-patients was 497, and the number of out-patients 5,337. The Chairman remarked that while the Governors must take steps to make the needs of the Children's Hospital widely known, no appeal would be issued, as it was the Derby Infirmary centenary. For personal details our readers are referred to our issue of December 17, page 366.

